# Investigation of the location effect of external markers in respiratory‐gated radiotherapy

**DOI:** 10.1120/jacmp.v9i2.2758

**Published:** 2008-04-16

**Authors:** Hui Yan, Guopei Zhu, James Yang, Mei Lu, Munther Ajlouni, Jae Ho Kim, Fang‐Fang Yin

**Affiliations:** ^1^ Department of Radiation Oncology Duke University Medical Center Durham North Carolina U.S.A.; ^2^ Department of Radiation Oncology Henry Ford Hospital Detroit Michigan U.S.A.; ^3^ Department of Radiation Oncology Cancer Hospital of Fudan University Shanghai China; ^4^ Department of Biostatistics Henry Ford Hospital Detroit Michigan U.S.A.

**Keywords:** External marker, tumor tracking, respiratory‐gated radiotherapy

## Abstract

In 7 lung and breast cancer patients, we investigated the location effect of external markers on the correlation between the motions of external markers and of an internal target under various breathing patterns.

Our department developed a tumor tracking system consisting of two infrared cameras and a medical simulator. Using the system, we monitored the simultaneous motions of tumor and external markers placed at various locations on a patient's skin and saved the results for offline analysis. We then used a cross‐covariance approach to analyze the correlation between the motions of individual markers and of the tumor. Based on the additive model, we evaluated the predictability of tumor motion from the motions of the external markers.

The effect of marker location on the correlation between the motions of the tumor and of the external markers varied widely from patient to patient. At no specific marker location did the surrogate signal consistently present superior correlation with tumor motion in 3 breathing sessions with 7 patients. When the composite external signal generated from multiple external motion signals was correlated with tumor motion, the quality of the correlation improved significantly. In most cases, the composite signal provided the best surrogate signal for correlating with tumor motion.

Correlation between the motions of external markers and of a tumor may be affected by several factors, including patient characteristics, marker locations, and breathing pattern. A single external marker cannot provide sufficient and reliable tracking information for tumor motion. A composite signal generated from the motions of multiple external makers provides an excellent surrogate signal, which in this study demonstrated superior correlation with tumor motion as compared with the signal provided by an individual marker. A composite signal would be a more reliable way to track tumor motion during respiratory‐gated radiotherapy.

PACS numbers: 87.53.Jw

## I. INTRODUCTION

Organ motion resulting from breathing, heartbeat, and normal metabolism contributes to the uncertainty of target localization in radiation therapy. In clinical practice, a properly defined margin is added to the gross target volume (GTV) to yield a planning target volume (PTV) that accounts for the uncertainty. The margin ensures that the GTV will receive the full dose prescribed, even if the PTV shifts to some extent from its planning location because of organ motion during the course of radiation therapy.

The issue of organ motion has drawn wide attention in cancer treatment, and accounting for motion is particularly important to cutting‐edge treatment techniques such as intensity‐modulated radiation therapy.^(^ ^1^ ^,^ ^2^ ^)^ When the GTV is extended to a PTV, certain regions of normal tissue are included in PTV and will potentially be treated with the full prescribed dose, possibly resulting in an increase in normal‐tissue complications. To reduce the risk caused by extension of the GTV, online target localization and tracking have to be enhanced in a direct or indirect manner.

Several breath‐hold techniques have been developed to minimize respiratory‐related organ motion during computed tomography scans and radiotherapy.^(^ ^2^ ^–^ ^9^ ^)^ The reproducibility of respiratory‐related organ motion can be improved with the appropriate application of breath control strategies.^(^ ^2^ ^–^ ^4^ ^)^ However, the efficacy of these techniques is limited by variability in patient's ability to maintain total lung capacity.

As reported by Shirato^(^ ^10^ ^–^ ^12^ ^)^ and Schweikard,^(^ ^13^ ^,^ ^14^ ^)^ direct tumor tracking systems have been employed in some institutions to compensate for organ motion during adaptive radiotherapy. These techniques provide the capability to track the tumor location using implanted metal seeds and markers together with a diagnostic X‐ray imaging system. However, continuous X‐ray imaging is necessary to track the internal target during the entire treatment period, and the total amount of radiation is significant—considerably limiting application of this technique in the clinic.

As an alternative, several indirect tumor tracking systems have been developed and adopted for clinical use. Examples include the spirometer and strain gauge, and external markers or sensors, such as infrared light‐emitting diodes.^(^ ^15^ ^–^ ^21^ ^)^ These techniques avoid the considerable fluoroscopic radiation exposure of direct tracking systems and allow patients to breathe freely while the tumor is continuously tracked. Nevertheless, success in implementing such systems relies on knowledge of the relationship between the external markers and the internal anatomy.

The typical use of diaphragmatic motion as a surrogate for the actual tumor is widely debated.^(22)^ Despite use of verbal instruction, the average diaphragm excursion at exhale is 3 mm (range: 2−5 mm), and at inhale, 7 mm (range: 4−10 mm), based on fluoroscopic examination. Variations in diaphragm position are also observed on repeat gated portal films.^(23)^ Because surrogate markers are randomly placed on the chest wall or abdomen to track or infer the motion of the tumor, optimal placement of those markers is critical and is still an unresolved question in clinical practice.

For the present study, we used a tumor‐monitoring system that was developed in‐house at our institution. It consists of two infrared cameras and a medical simulator, and is capable of simultaneously monitoring the motion of the internal target and of multiple external markers placed at various locations on patient's skin. Both types of motion signal are recorded on a hard disk in a digital format for offline analysis. We enrolled 7 patients undergoing radiation therapy for lung and breast cancers into our study protocol. On each patient's skin, 3−5 external markers were placed at given locations to serve as surrogates for tracking tumor motion. We used a cross‐correlation approach and an additive model to analyze correlations between the motions of the external markers and those of the internal target.

## II. MATERIALS AND METHODS

### A. Eligibility

The 7 patients enrolled in the study were undergoing radiation therapy for lung metastases (n=6) or breast cancer (n=1), right upper or middle lobe lesions; all had a Karnofsky performance status above 70. To be eligible for the study, each patient's tumor volumes had to be clearly and unambiguously identifiable from fluoroscopic images taken before the acquisition sequence. Visibility of the tumor mass (or implanted target) was checked in the anterior—posterior (AP) and lateral directions.

### B. Marker placement

The external markers were placed on each patient's chest at locations chosen by the physician. Generally, 4 infrared reflective markers were placed at the left upper lobe (LUL), above the tumor (AT), at the xiphoid (X), and at the right lower lobe (RLL). In some patients, a 5th marker was placed at right upper lobe (RUL). In some patients, only 3 markers (LUL, AT, and X) were used.

### C. Data acquisition

The custom‐developed tumor‐tracking system used for the present work consists of two infrared cameras and a medical simulator. The sampling signals of three devices were synchronized so as to acquire simultaneous surrogate signals of external and internal markers.

In brief, a simulator (Ximatron: Varian Medical Systems, Palo Alto, CA) takes a fluoroscopic video, which is sent to a video converter where the video stream is converted to a digital image series. The original video stream is forwarded to a monitor for display. The digital image series is forwarded to a frame grabber, where the images are re‐sampled based on the synchronization signal that accompanies the sampling signal from the infrared cameras. Thus, the fluoroscopic images in the frame grabber are synchronized with the locations of external markers as sampled by the infrared cameras. During offline analysis, the tumor center is identified and labeled on the fluoroscopic images by experienced clinicians using an image tool, and the results are exported to text files.

The infrared cameras were borrowed from a clinical patient positioning system (ExacTrac: BrainLAB, Feldkirchen, Germany). Two infrared cameras (MCU 120: Qualisys, Gothenburg, Sweden) were installed on the ceiling of the simulation room and were pointed toward the couch.

The sampling rate of the infrared cameras is relatively low, and it varies slightly with the number of external markers to be tracked. Detection of the external markers also varies with patient geometry and marker arrangement. Our experiments showed that ambiguity in detection is a possibility when more than 5 external markers are placed on the patient's skin.

The coordinates of the external markers (in three dimensions) were sampled at a frequency of approximately 8−10 Hz and were saved to a text file. In each acquisition sequence, we captured 3 time series corresponding to the coordinates of each external marker in each of three dimensions and 2 time series corresponding to the coordinates of the tumor in each of two dimensions.

The protocol to acquire the motion signals consisted of 3 sessions:

- **Session 1**—Record fluoroscopic video and external marker locations for approximately 30 – 40 s while the patient maintains free breathing.
- **Session 2**—Record free breathing for 10 s, and then change to breath‐holding for 5 s. Resume free breathing for another 10 s, and then breath‐holding for another 5 s. The patient then returns to free breathing.
- **Session 3**—Record fluoroscopic video and external marker locations for another approximately 30−40 seconds once the patient resumes free breathing after session 2.

The patient is given a break of approximately 20−30 seconds between two acquisition sequences. In the first sequence, the three breathing sessions are conducted while the patient is imaged with the gantry of the simulator rotated to 0 degrees (AP direction). The three breathing sessions are then repeated while the patient is imaged with the gantry of the simulator rotated to 270 degrees (right lateral direction).

### D. Data analysis

We normalized the time series by shifting their mean values to 0 and rescaling to a set range, [−1,1]. Because we had earlier observed phase shifts between the time series of the external markers and of the tumor motion,^(^ ^10^ ^,^ ^17^ ^)^ we used an automatic program to correct for those shifts. First, the peak locations in the time series of external and internal motions were automatically identified. If the shifts between the two series were consistent, the series (external motion and tumor motion) were then aligned without a further operation being conducted. In most cases, the phase shifts observed between the internal and external motion signals were constant, because the patients' breathing was highly reproducible and stable during the short acquisition time. If necessary, a cross‐covariance function was used to automatically detect and correct phase shift between the two time series.^(24)^ Once the phase shift was corrected, the correlation coefficient between the internal and external motion signals was calculated.

To investigate the effect of multiple markers on the predictability of tumor motion, we tested many statistical models, finally choosing the additive model. The additive model is popular in time series analysis because of its high predication accuracy and computational efficiency. It is especially suited to time series featuring a “seasonal pattern.”

We used additive models to establish the location relationship between tumor and markers^(25)^ as shown here:

(1) $$Y_{t}=\alpha +\sum_{m=1}^{M}s_{m}(X_{mt})+\varepsilon_{mt},$$

where $Y_{t}$ is the tumor location, $X_{mt}$ is *m*th marker location (m=1,…,M) at time t(t=1,…,T), $\varepsilon_{mt}$ is the error term, $S_{m}$ is a smooth function for the *m*th marker, and α is the average tumor location when the marker locations are set at baseline. The smooth function, $S_{m}$, is an arbitrary univariate function, one for each predictor. The smooth functions can be estimated using any scatter‐plot smoother. In the present study, we used a smoothing spline for estimating $S_{m}$.

The advantages of additive model are that each predictor is estimated using a univariate smoother so that the curse of dimensionality is avoided and that the prediction of tumor location on each individual marker depends on the marker location so that the precision of the tumor location prediction increases.

For each breathing session, we tested two kinds of prediction. First, we used the motion signal from an individual marker to attempt to predict tumor motion in the same dimension. We then used other markers for comparison to evaluate the accuracy of the prediction. Second, we used the motion signals from all of a patient's available markers to predict tumor motion in the same dimension. To evaluate the accuracy of the prediction, we compared those results with the results achieved using an individual marker for prediction.

All computations were handled by R program, a language for statistical computing and graphics that provides numerous statistical and graphics tools for the statistical community.^(26)^ We used $R^{2}$, the square of multiple correlation coefficients, to evaluate goodness of fit. The value of $R^{2}$ ranges between 0 and 1, representing the proportion of the variation in the response data (tumor motion) accounted for in the model. The higher the $R^{2}$ value, the better the fit to the response signal (tumor motion).

## III. RESULTS

**Figure 1:**
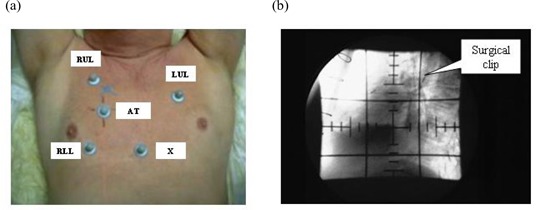
(a) An example of the arrangement of external skin markers on a patient. (b) Fluoroscopic image clearly showing the location of an implanted surgical clip used to represent the internal target.

Table 1 summarizes the characteristics of the study patients. The patients had no notable discomfort or difficulty in maintaining the treatment position during simulation. Fluoroscopic video of tumor motion and of motion of the associated external markers was captured by the simulator and the infrared cameras respectively. Fig. 1(a) shows an arrangement of 5 external markers placed on a patient's chest. Figure 1(b) shows the location of a surgical clip that was used to represent the internal target for one patient. In most cases, the center of the tumor, clearly visible on the fluoroscopic images, was used to represent the internal target.

**Figure 2:**
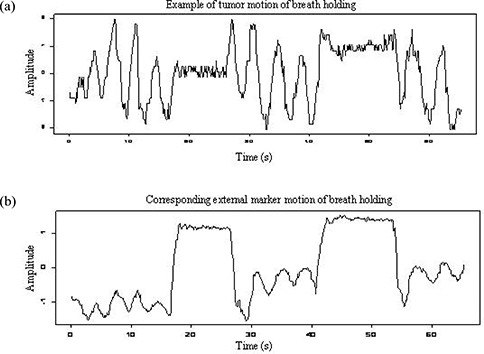
Example of the motion signals of an external marker and internal target for a patient in breathing session 2. (a) Trajectory of tumor motion in the lateral direction identified from fluoroscopic images acquired in the anterior—posterior view. (b) Trajectory of an external marker in the lateral direction recorded by infrared camera.

**Figure 3:**
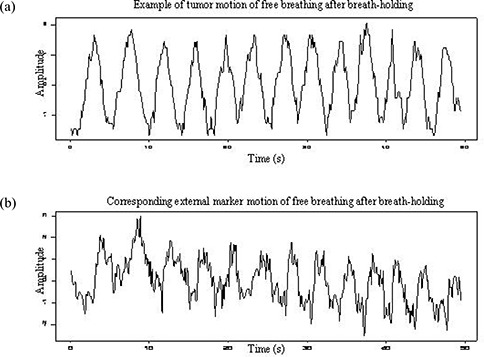
Example of the motion signals of an external marker and internal target for a patient in breathing session 3. (a) Trajectory of tumor motion in the lateral direction identified from fluoroscopic images acquired in the anterior—posterior view. (b) Trajectory of an external marker in lateral direction recorded by infrared camera.

Figs. 2 and 3 show a pair of motion trajectories for the internal target and external markers acquired in breathing sessions 2 and 3. A clear correspondence between them from cycle to cycle can be observed, but a certain discrepancy exists between the amplitudes and peak locations of the two signals.

Table 2 lists the calculated correlation coefficients between the marker and tumor motions in the 7 patients. Because multiple markers were used for each patient, Table 2 presents only the marker with the largest correlation coefficient in each dimension. The correlation coefficient varies from 0.247 to 0.987 for all sessions.

Several results can be observed from Table 2. First, the quality of the correlation provided by an individual marker is inconsistent from patient to patient. No single specific marker location constantly presents superior correlation with the internal target over all breathing sessions in the 7 patients. Second, the quality of the correlation is affected by marker location. Based on the averages of the correlation coefficients calculated at 5 locations, the mean values and standard deviations are 0.70±0.18 (LUL), 0.71±0.19 (AT), 0.55±0.20 (X), 0.69±0.22 (RLL), and 0.62±0.21 (RUL). The markers placed at LUL and AT showed the best correlation with tumor motion. The quality of correlation provided by the RLL location is closer to LUL and AT, but with increasing deviation. The quality of correlation provided by RUL and X is relatively low, but acceptable. Third, the quality of correlation is affected by breathing patterns. In 6 of the 7 patients, session 1 provides better correlations between the motions of the external markers and the internal target than does session 3. In 3 of the 7 patients, session 2 provides better correlations than do sessions 1 and 3; in another 3 patients, the opposite result is true.

**Table 1:** Summary of patient characteristics

*Patient*	*Age*	*Sex*	*Staging performance status*	*Karnofsky volume (cm^3^)*	*Target location*	*Lesion*	*Histology* (n)	*Markers*
1	76	Male	T1N0M0	80	7.52	RUL	Squamous cell carcinoma	3
2	76	Male	T2N0M0	70	13.12	RUL	Squamous cell carcinoma	5
3	75	Male	T1N0M0	70	8.14	RUL	Squamous cell carcinoma	5
4	73	Male	T2N0M0	70	30.24	RUL	Squamous cell carcinoma	4
5	58	Female	Unevaluated^a^	70	Clip	RUL	Adenocarcinoma^a^	4
6	75	Female	T1N0M0	80	1.69	RML	Unsure	4
7	72	Male	T2N0M0	70	34.75	RML	Squamous cell carcinoma	5

RUL=right upper lobe; RML=right middle lobe.

a Left breast cancer with metastases to the right middle lobe of lung

**Table 2:** Maximum correlation coefficient between motions of external marker and internal target in each dimension^a^

*Patient*	*Anterior—posterior view*						*Lateral view*					
	*Session 1*		*Session 2*		*Session 3*		*Session 1*		*Session 2*		*Session 3*	
	*Lat*	*Long*	*Lat*	*Long*	*Lat*	*Long*	*Vert*	*Long*	*Vert*	*Long*	*Vert*	*Long*
1	LUL	AT	LUL	AT	LUL	AT	X	AT	LUL	LUL	X	X
	0.47	0.89	0.42	0.79	0.32	0.73	0.58	0.88	0.92	0.90	0.76	0.67
2	LUL	LUL	X	LUL	RLL	RUL	AT	X	LUL	AT	AT	X
	0.40	0.42	0.65	0.63	0.37	0.90	0.74	0.35	0.57	**0.24**	0.31	0.37
3	RLL	LUL	X	RLL	LUL	RUL	LUL	AT	AT	AT	RLL	RLL
	0.74	0.85	0.36	0.51	0.93	**0.98**	0.85	0.61	0.66	0.55	0.84	0.56
4	LUL	RLL	AT	AT	X	LUL	X	RLL	RLL	LUL	X	X/RLL
	0.68	0.90	0.48	0.90	0.35	0.64	0.73	0.86	0.90	0.76	0.88	0.63
5	LUL	AT	RLL	RLL	LUL	X	RLL	LUL	RLL	X	RLL	AT
	0.57	0.50	0.37	0.44	0.73	0.30	0.80	0.82	0.41	0.45	0.78	0.88
6	LUL	X	AT	LUL	X	RLL	LUL	LUL	LUL	LUL	RLL	RLL
	0.71	0.26	0.68	0.51	0.77	0.37	0.84	0.83	0.82	0.78	0.85	0.72
7	X	AT	AT	RUL/AT	RLL	AT	LUL	RUL	RLL	RUL/AT	X	AT
	0.51	0.80	0.79	0.48	0.37	0.90	0.90	0.72	0.96	0.62	0.82	0.75

Lat=lateral; Long=longitudinal; Vert=vertical; LUL=left upper lobe; AT=above the tumor; X=xiphoid; RLL=right lower lobe; RUL=right upper lobe.

a Values in boldface type indicate the maximum and minimum of the correlation coefficients between the motions of the external marker and the internal target.

We used the additive model to investigate the predictability of target motion based on the motion of external markers. As a measure of the performance of the prediction, we determined $R^{2}$ values for all pairs of individual marker and tumor motions, and for the composite marker and tumor motions. The results are summarized in Tables 3 – 5, for the individual sessions 1, 2, and 3. In each table, the values of $R^{2}$ for the tumor motion prediction based on either individual markers or the composite makers are reported. For example, at session 1 for patient 3, the values of $R^{2}$ are 0.219 (LUL), 0.0194 (AT), 0.0202 (X), 0.0271 (RLL), and 0.123 (RUL) when an individual marker was used for predicting tumor motion in the lateral direction. In the model established based on the composite markers, the value of $R^{2}$ is 0.349.

To investigate the ability of external markers to detect unexpected respiratory motion (a cough or a breath interruption for any reason, for instance), two breathing‐hold intervals were designed into session 2. Session 3, immediately following, used free breathing. Compared with the motion trajectory shown in Fig. 2(b), the trajectory in Fig. 3(b) shows a “noisier” signal and a tendency to shift its baseline away from 0. Similar results were observed for patients 1, 2, 3, and 6.

## IV. DISCUSSION

The results presented here are based on motion signals from external markers and an internal target acquired for each of 7 patients. Although details varied between the patients, some features were common. No patient had difficulty controlling his or her breathing according to the clinician's instruction, and all lesions were located in an anatomic area (right upper and middle lobes) that is less affected by the heartbeat and is clearly visible under fluoroscopy. The amplitude of respirator movement of lung tumors in the upper lobe was estimated to be as little as 6.2 mm (range: 2.4−11.3 mm) in the craniocaudal direction and 2.2 mm (range: 0−6 mm) in the AP direction.^(27)^ Comparatively, tumor movement in the lower lobe was found to be 9.1 mm (range: 3.4−24.0 mm) in the craniocaudal direction, and 10.1 mm (range: 0−22 mm) in the AP direction.^(28)^

From the correlation analysis, the motion characteristics of the internal and external markers varied dimension by dimension, session by session, and patient by patient. No single marker location consistently provided superior correlation with the target motion over all breathing sessions. Considering that breathing is a process of elastic volume compression and expansion forced by a semi‐rigid thoracic cage and a non‐rigid diaphragm, various regions inside the chest or adjacent to the chest wall may move with inconsistent properties. For a tumor located at a deep or shallow location inside a patient's chest or close to the diaphragm, differences in breath motion could be more significant. That finding is consistent with previous reports by Ozhasoglu,^(22)^ Bruce,^(29)^ Donaldson,^(30)^ and Benchetrit.^(31)^

Respiratory motion is the most difficult situation in gating studies, given that breathing motions are less predicable and never stationary. The breathing patterns observed in one patient or one breathing session cannot be assumed to be highly reproducible in another patient or session.

Compared with the correlation performance of an individual marker, a composite signal generated from the motions of multiple markers using the additive model presents a superior surrogate signal for tumor tracking. On average, the value of $R^{2}$ was improved by 56.1% with the use of a composite signal. That finding implies that a composite signal might improve the reliability and accuracy of tumor tracking using external markers.

**Table 3:** Values of $R^{2}$ evaluating goodness of fit in using the additive model to correlate individual marker or composite marker motion with the internal target motion in session 1

*Patient*	*View*	*Dim*	$M_{LUL}$ $M_{LUL}$	$M_{AT}$ $M_{AT}$	$M_{X}$ $M_{X}$	$M_{RLL}$	$M_{RUL}$	$M_{COMP}$
1	AP	Lat	0.0062	0.0088	−0.0003	—	—	0.0758
		Long	0.1860	0.0410	0.0282	—	—	0.6220
	Lateral	Vert	0.0389	0.1950	0.2110	—	—	0.3140
		Long	0.7830	0.7330	0.7550	—	—	0.8040
2	AP	Lat	0.0686	0.2430	0.0134	0.0036	0.0381	0.4110
		Long	0.0219	0.0220	0.0799	0.0731	0.0845	0.2170
	Lateral	Vert	0.0965	0.1270	0.4360	0.2210	0.3970	0.6960
		Long	0.4960	0.4480	0.1850	0.4350	0.1780	0.7270
3	AP	Lat	0.2190	0.0194	0.0202	0.0271	0.1230	0.3430
		Long	0.1100	0.2280	0.3290	0.3230	0.3240	0.8090
	Lateral	Vert	0.8210	0.3820	0.7490	0.4090	0.3780	0.8830
		Long	0.1540	0.5700	0.2870	−0.0019	0.0805	0.7960
4	AP	Lat	0.1040	0.0826	0.1200	0.1250	—	0.4330
		Long	0.0642	0.0597	0.0677	0.0636	—	0.1450
	Lateral	Vert	0.2740	0.2720	0.1610	0.1800	—	0.7900
		Long	0.6010	0.6010	0.3160	0.6280	—	0.7000
5	AP	Lat	0.0062	0.0088	−0.0003	0.0526	—	0.0758
		Long	0.1860	0.0410	0.0282	0.2290	—	0.6220
	Lateral	Vert	0.0389	0.1950	0.2110	0.2340	—	0.3140
		Long	0.7830	0.7330	0.7550	0.7820	—	0.8040
6	AP	Lat	−0.0015	0.0161	0.0169	0.0414	—	0.0943
		Long	0.7700	0.7290	0.7070	0.0786	—	0.8660
	Lateral	Vert	0.0237	0.0320	0.1020	−0.0022	—	0.3020
		Long	0.0390	0.0915	0.1370	0.0540	—	0.6460
7	AP	Lat	0.0265	0.1170	0.0133	0.0447	0.0469	0.1640
		Long	0.0676	0.0312	0.0310	0.0483	0.0655	0.1960
	Lateral	Vert	0.0498	0.5100	0.4630	0.1490	0.4350	0.6290
		Long	0.1210	0.1620	0.1620	0.0745	0.0650	0.2710

$M_{LUL}=marker\;motion\;at\;left\;upperlobe$; $M_{AT}=marker\;motion\;above\;tumor$; $M_{X}=marker\;motion\;at\;xiphoid$; $M_{RLL}=marker\;motion\;at\;right\;lower\;lobe$; $M_{RUL}=marker\;motion\;at\;right\;upper\;lobe$; $M_{COMP}=composite\;marker\;motion$; AP=anterior−posterior; Lat=lateral; Long=longitudinal; Vert=vertical.

**Table 4:** Values of $R^{2}$ evaluating the goodness of fit in using the additive model to correlate individual marker or composite marker motion with internal target motion in session 2

*Patient*	*View*	*Dim*	$M_{LUL}$ $M_{LUL}$	$M_{AT}$ $M_{AT}$	$M_{X}$ $M_{X}$	$M_{RLL}$	$M_{RUL}$	$M_{COMP}$
	AP	Lat	0.1160	0.1420	0.0784	—	—	0.3250
		Long	0.5820	0.5670	0.5950	—	—	0.7570
	Lateral	Vert	0.7120	0.6190	0.7210	—	—	0.8700
		Long	0.5790	0.5270	0.5020	—	—	0.7460
2	AP	Lat	0.0295	0.0842	0.0601	0.1230	0.1100	0.3190
		Long	0.0364	0.0321	0.0250	0.0326	0.0222	0.0866
	Lateral	Vert	0.0985	0.1500	0.0343	0.0805	0.0348	0.2330
		Long	0.0937	0.0843	0.1430	0.1260	0.1540	0.3810
3	AP	Lat	0.2740	0.5180	0.3210	0.3780	0.4130	0.6830
		Long	0.2290	0.3740	0.2400	0.1970	0.1950	0.6130
	Lateral	Vert	0.9110	0.770	0.9440	0.9190	0.9240	0.9760
		Long	0.4700	0.5780	0.4460	0.3610	0.3770	0.8320
4	AP	Lat	0.2130	0.4500	0.6320	0.1290	—	0.7810
		Long	0.4540	0.4290	0.1340	0.3880	—	0.4990
	Lateral	Vert	0.1820	0.2620	0.2560	0.2390	—	0.4410
		Long	0.4750	0.4890	0.0859	0.5770	—	0.7730
5	AP	Lat	0.1160	0.1420	0.0784	0.0393	—	0.3250
		Long	0.5820	0.5670	0.5950	0.6430	—	0.7570
	Lateral	Vert	0.7120	0.6190	0.7210	0.6580	—	0.8700
		Long	0.5790	0.5270	0.5020	0.5430	—	0.7460
6	AP	Lat	0.1300	0.3190	0.0753	0.0760	—	0.4620
		Long	0.0437	0.1050	0.0968	0.1160	—	0.4940
	Lateral	Vert	0.8730	0.8300	0.5940	0.8060	—	0.8980
		Long	0.0695	0.0612	0.3090	0.2960	—	0.8820
7	AP	Lat	0.1200	0.2260	0.2890	0.1160	0.0613	0.4660
		Long	0.0984	0.1300	0.0938	0.1150	0.1230	0.3080
	Lateral	Vert	0.0299	0.0063	−0.0018	0.0216	0.0625	0.1980
		Long	0.1380	0.1590	0.1800	0.1260	0.1160	0.4450

$M_{LUL}=marker\;motion\;at\;left\;upperlobe$; $M_{AT}=marker\;motion\;above\;tumor$; $M_{X}=marker\;motion\;at\;xiphoid$; $M_{RLL}=marker\;motion\;at\;right\;lower\;lobe$; $M_{RUL}=marker\;motion\;at\;right\;upper\;lobe$; $M_{COMP}=composite\;marker\;motion$; AP=anterior−posterior; Lat=lateral; Long=longitudinal; Vert=vertical.

**Table 5:** Values of $R^{2}$ evaluating the goodness of fit in using the additive model to correlate individual marker or composite marker motion with internal target motion in session 3

*Patient*	*View*	*Dim*	$M_{LUL}$ $M_{LUL}$	$M_{AT}$ $M_{AT}$	$M_{X}$ $M_{X}$	$M_{RLL}$	$M_{RUL}$	$M_{COMP}$
1	AP	Lat	0.0654	0.0989	0.1020	—	—	0.1340
		Long	0.3990	0.3460	0.3140	—	—	0.6130
	Lateral	Vert	0.4780	0.4890	0.5240	—	—	0.7220
		Long	0.4070	0.3380	0.4440	—	—	0.5470
2	AP	Lat	0.0701	0.3440	0.1010	0.0342	0.3510	0.5200
		Long	−0.0034	0.0215	0.0130	−0.0083	0.0085	0.1310
	Lateral	Vert	0.3230	0.1520	0.5380	0.1050	0.6090	0.6920
		Long	0.3850	0.4430	0.1080	0.2560	0.1320	0.8020
3	AP	Lat	0.1150	0.0292	0.0913	0.1030	0.0544	0.4480
		Long	0.3170	0.4540	0.3540	0.1130	0.4010	0.8330
	Lateral	Vert	0.7600	0.2050	0.6980	0.3360	0.3300	0.8240
		Long	0.1180	0.3450	0.0521	0.2620	0.4010	0.7870
4	AP	Lat	0.0866	0.0712	0.0645	0.5290	—	0.6350
		Long	0.1080	0.1140	0.0874	0.1170	—	0.1390
	Lateral	Vert	0.4990	0.7840	0.2930	0.6860	—	0.8510
		Long	0.6550	0.6140	0.5290	0.6990	—	0.7620
5	AP	Lat	0.0654	0.0989	0.1020	0.0638	—	0.1340
		Long	0.3990	0.3460	0.3140	0.2360	—	0.6130
	Lateral	Vert	0.4780	0.4890	0.5240	0.4930	—	0.7220
		Long	0.4070	0.3380	0.4440	0.4150	—	0.5470
6	AP	Lat	0.2700	0.0940	0.3860	0.3680	—	0.4800
		Long	0.7510	0.7910	0.8280	0.7850	—	0.8680
	Lateral	Vert	0.3860	0.4700	0.0978	0.2650	—	0.6050
		Long	0.0457	0.0671	0.0409	0.8380	—	0.8790
7	AP	Lat	0.3130	0.2190	0.0126	0.0282	0.0732	0.4670
		Long	0.0084	0.0137	0.0281	0.0051	−0.0006	0.0501
	Lateral	Vert	0.0461	0.3670	0.3930	0.1070	0.0876	0.4270
		Long	0.0723	0.0826	0.0936	0.0215	0.0713	0.1150

$M_{LUL}=marker\;motion\;at\;left\;upperlobe$; $M_{AT}=marker\;motion\;above\;tumor$; $M_{X}=marker\;motion\;at\;xiphoid$; $M_{RLL}=marker\;motion\;at\;right\;lower\;lobe$; $M_{RUL}=marker\;motion\;at\;right\;upper\;lobe$; $M_{COMP}=composite\;marker\;motion$; AP=anterior‐posterior; Lat=lateral; Long=longitudinal; Vert=vertical.

The AT external marker showed the best performance in most of cases that we investigated. No individual X marker demonstrated excellent correlation with the internal target in our study, although some investigators have reported that the motion of a marker placed at the xiphoid correlates well with diaphragmatic motion.^(^ ^32^ ^,^ ^33^ ^)^ However, generalization of the foregoing conclusion is limited, because we observed few cases in which the AT marker did not present a better correlation than that provided by the X marker. Based on correlation and predictability analysis, our findings lead to a recommendation of marker placement at LUL, RLL, and AT. Those sites are the most common at which to place external makers in clinical practice. Although we do not know which arrangement of markers would provide the best correlation output, our study shows that a composite signal generated from the motions of multiple markers has the potential to improve the predictability of tumor motion.

We also investigated breath‐holding and its effect on a subsequent free‐breathing session. Non‐stationary motion was observed during session 3 in most instances. The ability of a patient to hold his or her breath for a short period is not a big issue, as demonstrated by the plateau in the motion trajectory shown in Fig. 2. However, the signals acquired in the subsequent free‐breathing session became irregular, which makes tumor tracking more difficult. When the patient's normal breathing is interrupted, the target may stop at a position away from its regular position and then resume moving from there. Phase shift and baseline migration could continue until the steady state is restored. Under those circumstances, the ability to predict target motion from surface marker motion could be completely lost. We noticed that after interruption of a free breathing pattern by breath‐holding, it took approximately 1−2 minutes for the free‐breathing pattern to be restored.

Modern radiotherapy can localize a rigid target and deliver a high dose to the volume with an accuracy of a few millimeters or even better. Management of target motion is therefore critical for treatment of lesions in the thoracic and abdominal regions. Not only should the patient's breathing be monitored, but the target motion associated with that breathing must also be tracked for the entire time. Diagnostic radiographs are helpful in determining the position of a tumor at beginning of treatment, but they are not useful during the treatment course. Continuously acquiring real‐time target positions during treatment is prohibited because of the associated high skin dose. Considering that breathing is a complex process that is only partially under conscious control and that is accompanied by involuntary actions such as coughing, assistance from a secondary, non‐ionizing radiation monitoring system is necessary for determining real‐time target motion. Our tracking system can be of great help, with several infrared sensors attached to the patient's skin and online target motion being tracked through composite marker motion. Such a system effectively detects respiration motion during treatment, and a rapid intervention—for example, switching off the beam—can be applied in case of unwanted movements of the tumor.

## V. CONCLUSION

We conducted a study to investigate the correlation between multiple surface markers and internal target motion, attempting to determine whether a specific maker location consistently correlates with tumor motion. Our study showed that no such location exists. A composite signal generated from the motion of multiple markers provides a surrogate signal that yields improved correlation with internal target motion. For radiation therapy applications using multiple surface markers and respiratory gating, our results suggest that a composite signal derived from the motions of all available external markers (instead of a surrogate signal provided by one marker) would be a reliable way to track a tumor.

## ACKNOWLEDGMENTS

We thank Archie Chu, PhD, at Henry Ford Hospital and Stephan Erbel at BrainLAB for technical support in setting up the infrared camera system. We also thank Robert Jones at Henry Ford Hospital for his clinical support in data acquisition.

## References

[1] Ohara K , Okumura T , Akisada M , et al. Irradiation synchronized with respiration gate. Int J Radiat Oncol Biol Phys. 1989; 17 (4): 853–857. 2777676 10.1016/0360-3016(89)90078-3

[2] Wong JW , Sharpe MB , Jaffray DA , et al. The use of active breathing control (ABC) to reduce margin for breathing motion. Int J Radiat Oncol Biol Phys. 1999; 44 (4): 911–919. 10386650 10.1016/s0360-3016(99)00056-5

[3] Stromberg JS , Sharpe MB , Kim LH , et al. Active breathing control (ABC) for Hodgkin's disease: reduction in normal tissue irradiation with deep inspiration and implications for treatment. Int J Radiat Oncol Biol Phys. 2000; 48 (3): 797–806. 11020577 10.1016/s0360-3016(00)00681-7

[4] Dawson LA , Brock KK , Kazanjian S , et al. The reproducibility of organ position using active breathing control (ABC) during liver radiotherapy. Int J Radiat Oncol Biol Phys. 2001; 51 (5): 1410–1421. 11728702 10.1016/s0360-3016(01)02653-0

[5] Hanley J , Debois MM , Mah D , et al. Deep inspiration breath‐hold technique for lung tumors: the potential value of target immobilization and reduced lung density in dose escalation. Int J Radiat Oncol Biol Phys. 1999; 45 (3): 603–611. 10524412 10.1016/s0360-3016(99)00154-6

[6] Rosenzweig KE , Hanley J , Mah D , et al. The deep inspiration breath‐hold technique in the treatment of inoperable non‐small‐cell lung cancer. Int J Radiat Oncol Biol Phys. 2000; 48 (1): 81–87. 10924975 10.1016/s0360-3016(00)00583-6

[7] Mah D , Hanley J , Rosenzweig KE , et al. Technical aspects of the deep inspiration breath‐hold technique in the treatment of thoracic cancer. Int J Radiat Oncol Biol Phys. 2000; 48 (4): 1175–1185. 11072177 10.1016/s0360-3016(00)00747-1

[8] Sixel KE , Aznar MC , Ung YC . Deep inspiration breath hold to reduce irradiated heart volume in breast cancer patients. Int J Radiat Oncol Biol Phys. 2001; 49 (1): 199–204. 11163515 10.1016/s0360-3016(00)01455-3

[9] Kim DJ , Murray BR , Halperin R , Roa WH . Held‐breath self‐gating technique for radiotherapy of non‐small‐cell lung cancer: a feasibility study. Int J Radiat Oncol Biol Phys. 2001; 49 (1): 43–49. 11163496 10.1016/s0360-3016(00)01372-9

[10] Shirato H , Shimizu S , Kunieda T , et al. Physical aspects of a real‐time tumor‐tracking system for gated radiotherapy. Int J Radiat Oncol Biol Phys. 2000; 48 (4): 1187–1195. 11072178 10.1016/s0360-3016(00)00748-3

[11] Minohara S , Kanai T , Endo M , Noda K , Kanazawa M . Respiratory gated irradiation system for heavy‐ion radiotherapy. Int J Radiat Oncol Biol Phys. 2000; 47 (4): 1097–1103. 10863083 10.1016/s0360-3016(00)00524-1

[12] Shimizu S , Shirato H , Ogura S , et al. Detection of lung tumor movement in real‐time tumor‐tracking radiotherapy. Int J Radiat Oncol Biol Phys. 2001; 51 (2): 304–310. 11567803 10.1016/s0360-3016(01)01641-8

[13] Schweikard A , Shiomi H , Adler J . Respiration tracking in radiosurgery. Med Phys. 2004; 31 (10): 2738–2741. 15543778 10.1118/1.1774132

[14] Schweikard A , Glosser G , Bodduluri M , Murphy MJ , Adler JR . Robotic motion compensation for respiratory movement during radiosurgery. Comput Aided Surg. 2000; 5 (4): 263–277. 11029159 10.1002/1097-0150(2000)5:4<263::AID-IGS5>3.0.CO;2-2

[15] Kubo HD , Hill BC . Respiration gated radiotherapy treatment: a technical study. Phys Med Biol. 1996; 41 (1): 83–91. 8685260 10.1088/0031-9155/41/1/007

[16] Kubo HD , Len PM , Minohara S , Mostafavi H . Breathing‐synchronized radiotherapy program at the University of California Davis Cancer Center. Med Phys. 2000; 27 (2): 346–353. 10718138 10.1118/1.598837

[17] Vedam SS , Keall PJ , Kini VR , Mohan R . Determining parameters for respiration‐gated radiotherapy. Med Phys. 2001; 28 (10): 2139–2146. 11695776 10.1118/1.1406524

[18] Keall PJ , Kini VR , Vedam SS , Mohan R . Motion adaptive X‐ray therapy: a feasibility study. Phys Med Biol. 2001; 46 (1): 1–10. 11197664 10.1088/0031-9155/46/1/301

[19] Berbeco RI , Nishioka S , Shirato H , Chen GT , Jiang SB . Residual motion of lung tumors in gated radiotherapy with external respiratory surrogates. Phys Med Biol. 2005; 50 (16): 3655–3667. 16077219 10.1088/0031-9155/50/16/001

[20] Koch N , Liu HH , Starkschall G , et al. Evaluation of internal lung motion for respiratory‐gated radiotherapy using MRI: part I—correlating internal lung motion with skin fiducial motion. Int J Radiat Oncol Biol Phys. 2004; 60 (5): 1459–1472. 15590177 10.1016/j.ijrobp.2004.05.055

[21] Hoisak JD , Sixel KE , Tirona R , Cheung PC , Pignol JP . Correlation of lung tumor motion with external surrogate indicators of respiration. Int J Radiat Oncol Biol Phys. 2004; 60 (4): 1298–1306. 15519803 10.1016/j.ijrobp.2004.07.681

[22] Ozhasoglu C , Murphy MJ . Issues in respiratory motion compensation during external‐beam radiotherapy. Int J Radiat Oncol Biol Phys. 2002; 52 (5): 1389–1399. 11955754 10.1016/s0360-3016(01)02789-4

[23] Mageras GS , Fuks Z , Leibel SA , et al. Computerized design of target margins for treatment uncertainties in conformal radiotherapy. Int J Radiat Oncol Biol Phys. 1999; 43 (2): 437–445. 10030273 10.1016/s0360-3016(98)00386-1

[24] Yan H , Yin FF , Zhu GP , Ajlouni M , Kim JH . The correlation evaluation of a tumor tracking system using multiple external markers. Med Phys. 2006; 33 (11): 4073–4084. 17153387 10.1118/1.2358830

[25] Hastie T , Tibshirani R . Generalized additive models. Chapman and Hall, 1990. 10.1177/096228029500400302 8548102

[26] The R Development Core Team . R: a language and environment for statistical computing. Reference index. R Foundation for Statistical Computing; 2006 [Available online at: www.et.bs.ehu.es/soft/fullrefman.pdf; cited 20 February 2008]

[27] Ross CS , Hussey DH , Pennington EC , Stanford W , Doornbos JF . Analysis of movement of intrathoracic neoplasms using ultrafast computerized tomography. Int J Radiat Oncol Biol Phys. 1990; 18 (3): 671–677. 2318701 10.1016/0360-3016(90)90076-v

[28] Shimizu S , Shirato H , Kagei K , et al. Impact of respiratory movement on the computed tomographic images of small lung tumors in three‐dimensional (3D) radiotherapy. Int J Radiat Oncol Biol Phys. 2000; 46 (5): 1127–1133. 10725622 10.1016/s0360-3016(99)00352-1

[29] Bruce EN . Temporal variations in the pattern of breathing. J Appl Physiol. 1996; 80 (4): 1079–1087. 8926229 10.1152/jappl.1996.80.4.1079

[30] Donaldson GC . The chaotic behaviour of resting human respiration. Respir Physiol. 1992; 88 (3): 313–321. 1615228 10.1016/0034-5687(92)90005-h

[31] Benchetrit G . Breathing pattern in humans: diversity and individuality. Respir Physiol. 2000; 122 (2–3): 123–129. 10967339 10.1016/s0034-5687(00)00154-7

[32] Mageras GS , Yorke E , Rosenzweig K , et al. Fluoroscopic evaluation of diaphragmatic motion reduction with a respiratory gated radiotherapy system. J Appl Clin Med Phys. 2001; 2 (4): 191–200. 11686740 10.1120/jacmp.v2i4.2596 PMC5726007

[33] Kini VR , Vedam SS , Keall PJ , et al. A dynamic non‐invasive technique for predicting organ motion in respiratory‐gated radiotherapy of the chest [Abstract]. Int J Radiat Oncol Biol Phys. 2001; 51 (3): 25–26.

